# Short report: Evaluation of wider community support for a neurodiversity teaching programme designed using participatory methods

**DOI:** 10.1177/13623613231211046

**Published:** 2023-11-09

**Authors:** Reesha Zahir, Alyssa M. Alcorn, Sarah McGeown, Will Mandy, Dinah Aitken, Fergus Murray, Sue Fletcher-Watson

**Affiliations:** 1 University of Edinburgh, UK; 2 University College London, UK; 3 Salvesen Mindroom Centre, UK; 4 Autistic Mutual Aid Society Edinburgh (AMASE), UK

**Keywords:** education, neurodiversity, participatory methods

## Abstract

Children with neurodevelopmental diagnoses often experience discrimination from their peers at school. This may result from a lack of understanding, and intolerance of differences in their thinking, communication and social interactions. Learning About Neurodiversity at School (LEANS) is a teaching programme designed to educate primary school children about the concept of neurodiversity. The LEANS programme was created by a neurodiverse team, using participatory methods. In the current study, we evaluated whether the wider neurodiverse community endorsed the planned design generated by our participatory approach. Respondents (*n* = 111) rated their support for key elements of the planned LEANS content, via an online survey. Participants were majority neurodivergent (70%), 98% of whom reported moderate-to-high familiarity with neurodiversity concepts. Over 90% of respondents expressed support for the planned content presented, and 73% of respondents endorsed the draft neurodiversity definition provided. A small number of respondents provided open-ended comments giving further detail on their views. Overall, the LEANS programme plan received a high level of support from this independent, neurodiversity-aware sample – demonstrating the potential of small-group participatory methods to generate wider community support. The completed resource is now available as a free online download.

**Lay abstract:**

Children with diagnoses such as autism, attention-deficit/hyperactivity disorder (ADHD), dyslexia and so on often experience bullying at school. This group can be described as *neurodivergent*, meaning they think and process information differently from most people. Previous research suggests that increasing people’s knowledge can be an effective way to reduce stigma and bullying. Therefore, we decided to create a primary school resource to teach about *neurodiversity* – the concept that all humans vary in how our brains work. Working with educators, our research team – which included neurodivergent people – developed plans for a teaching programme called Learning About Neurodiversity at School (LEANS). Next, we wanted to know whether these plans, developed by our small neurodiverse team, would be endorsed by the wider community. To find out, we conducted an online feedback survey about our plans for the resource. We analysed feedback from 111 people who participated. Most of them identified as neurodivergent (70%) and reported being familiar with neurodiversity (98%), meaning they could provide an informed opinion on our plans. Over 90% of people expressed support for the planned programme content described in the survey, and 73% of them approved our intended definition of the resource’s core concept, *neurodiversity.* From these results, we concluded that there was a high level of support for the planned LEANS programme content across those from the wider community who completed the survey. Consequently, we continued developing the LEANS programme in line with the initial plans from our neurodiverse team. The completed resource is now available as a free download.

Children with neurodevelopmental diagnoses such as autism, attention-deficit/hyperactivity disorder (ADHD) and dyslexia face a number of adverse outcomes at school, including higher rates of peer victimisation, increased school exclusion and poorer mental health compared to peers without such diagnoses (Øksendal et al., 2019; Fleming et al., 2020; Hansen et al., 2018; Paget et al., 2015). One likely reason for this is a lack of awareness and misconceptions about neurodevelopmental conditions in school communities (Campbell & Barger, 2011; Gini et al., 2021). This can drive negative attitudes and intolerance of differences related to these groups (Moldavsky & Sayal, 2013; Turnock et al., 2022).

Increasing awareness is a well-established approach to improve acceptance, and several studies have demonstrated the effectiveness of educational interventions in improving attitudes towards people belonging to a specific diagnostic group, such as autism (Engel & Sheppard, 2020; Jones et al., 2021; Morton & Campbell, 2008). However, this is inefficient in the context of an inclusive mainstream classroom which may contain children with a number of different diagnoses plus those who are not yet diagnosed, but are unlikely to have a large number of pupils in any one diagnostic group (Gillooly & Riddell, 2019). In addition, pupils may hesitate to disclose a neurodevelopmental diagnosis such as autism due to perceived negative outcomes and stigma, making interventions that focus on a specific diagnostic label ineffective in this context (Thompson-Hodgetts et al., 2020). Instead, we developed a whole-class programme to teach about differences between people which result from neurodiversity, in an effort to improve knowledge and attitudes.

The Learning About Neurodiversity at School (LEANS) programme is a free, downloadable, teacher-delivered curriculum (15–19-hour classroom delivery time) designed to educate primary school children (aged 8–11 years) about the concept of neurodiversity using a mix of hands-on activities, storytelling items, and other resources (Alcorn et al., 2022). The neurodiversity framework is particularly useful for teaching about neurodevelopmental differences, as it conceptualises information processing differences in the brain as part of natural human variation (Masataka, 2017). Neurodivergence is when these differences substantially diverge from the dominant societal standard considered normal, or neurotypical (Walker, 2021). These differences underpin diagnostic labels such as autism, ADHD, dyspraxia and so on. The LEANS programme highlights the experiences of neurodivergent pupils in the classroom and how people can be more understanding and accommodating of their own and others’ needs.

Using a participatory design approach in educational contexts (e.g. developing curricula and designing learning environments) is thought to provide many benefits, ranging from improved learning effectiveness to increased uptake and utilisation of new educational products (Pnevmatikos et al., 2020). Therefore, the LEANS programme was designed using participatory methods. A neurodiverse team of eight educators from the United Kingdom and Republic of Ireland worked with the research team in an iterative participatory design process to develop LEANS’ educational content, format, and structure. However, a general concern with the participatory design approach is that views from a small, well-defined group of stakeholders may not always be upheld by the wider, more diverse community of people who will engage with the outputs of the process (Brereton & Buur, 2008). We attempted to address this concern in the second stage of LEANS programme development, which focused on getting views from multiple stakeholder groups on the acceptability and perceived usefulness of the planned resource before finalising and trialling the materials.

The current study was conducted as part of this consultation stage. We aimed to evaluate to what extent the plans for key elements of the programme (which came from our small, neurodiverse design team) were endorsed by a larger, independent group of stakeholders. LEANS is a whole-class resource for mainstream schools, which means there will be a mix of neurodivergent *and* neurotypical adult and child users. The programme is unlikely to achieve its educational aims unless it is broadly acceptable across a range of stakeholders. Therefore, we aimed to consult stakeholders who were likely to engage with the curriculum or be affected by its outcomes, that is both neurodivergent and neurotypical people, particularly educators and parents. To achieve these aims, we used an online survey to consult with an independent, neurodiverse sample.

## Methods

### Participants

Participants were adults (18 years or older) who responded to social media advertising. There were no inclusion and exclusion criteria, but we did recruit via neurodiversity-aware channels (such as neurodiversity-focused Facebook groups and charity mailing lists).

Complete demographic information and sample characteristics are detailed in Table 1. The sample was majority female (79%) and white (92%), and the mean age was 39 years (*SD* = 10.5). Half of the respondents reported experience working in education, and a similar proportion were parents/carers of a child aged under 18 years (46%). Furthermore, 98% of respondents reported that they were ‘somewhat’ to ‘very’ familiar with the concept of neurodiversity. Seventy per cent of the sample reported being neurodivergent, and 77% reported having at least one neurodivergent family member. The majority of neurodivergent respondents reported having multiple, overlapping neurodivergent diagnoses, with autistic spectrum diagnoses being the most common.

**Table 1.** Demographic information and sample expertise characteristics.

*Whole sample characteristics*	
Total *n*	111
Geographical location *n*	
England	58
Northern Ireland	3
Scotland	25
Wales	1
Ireland	5
USA	14
Other ^a^	5
Age in years, mean (*SD*)	39 (10.5)
Gender (%) – Male:Female:Other	13:79:8
Ethnicity (%) – Asian:Mixed:White ^b^	3:5:92
> 2 years experience in an educational role *n* (%)	55 (50)
Parents/carers *n* (%)	51 (46)
Rated familiarity with neurodiversity (%) – Unfamiliar:Somewhat:Very familiar	2:13:85
Neurodivergent *n* (%) ^c^	74 (67)
Reported diagnoses *n* ^c^	
Autism spectrum disorder	55
Attention-deficit/hyperactivity disorder	41
Asperger’s syndrome	32
Pervasive development disorder – not otherwise specified	3
Dyslexia	20
Dyspraxia	12
Developmental language disorder	2
Other	9
Has at least one neurodivergent family member *n* (%)	85 (77)

a Other locations included Australia, Germany and Canada.

b All ethnicity groups from the standard UK census were listed as response options in the survey.

c Includes formally diagnosed, suspected and self-identifying participants.

### Materials

The survey was delivered via the online platform Qualtrics. A copy of the full survey can be found in the supplementary materials (Appendix I). The first section of the survey collected characteristics required to describe the sample. These included being neurodivergent, having experience working in education, being a parent/carer of a child aged under 18 years and having familiarity with the concept of neurodiversity.

The second section of the survey asked respondents to rate our plans for the LEANS programme based on aspects such as the planned goals of the programme, its structure and content. In this brief report, we will selectively focus on key resource components that concern the goals and content of the LEANS programme. We do not report on evaluations of structure, not least because the preliminary structure presented in this survey changed substantially before the resource was completed, making this feedback less materially relevant. These key planned resource components include: (1) a brief description of the LEANS programme; (2) the planned definition of neurodiversity to be used in the programme; (3) a list of the planned goals of the LEANS programme; and (4) the learning objectives for each of the seven educational units that constitute the LEANS curriculum. A descriptive summary of these components can be found in Table 2.

**Table 2.** Summary of key planned LEANS resource components included in the online survey.

Planned resource component	Description
Resource description	This included information about what the LEANS programme is, the purpose of the programme, and who it is intended for
Resource goals	1: To increase knowledge of neurodiversity terms and concepts 2: To increase individuals’ positive and inclusive actions within the school community 3: To create more positive attitudes towards neurodiversity and neurodivergent people
Neurodiversity definition	“Neurodiversity is the fact that all human beings vary in the way our brains work. We take in information in different ways, we process it in different ways, and thus we behave in different ways. Neurodiversity is a property of the entire human race – each individual person is different from the next. Neurodiversity also gives rise to categorical differences between people. These categorical differences in brain processes, and therefore in experiences and behaviour, underpin diagnostic labels such as autism or dyspraxia”
Unit 1 ‘Introduction to neurodiversity’ learning objectives	Learning objectives addressed neurodiversity concepts and terminology
Unit 2 ‘Learning and thinking differently’ learning objectives	Learning objectives addressed the variability of experiences with lessons and the physical school environment
Unit 3 ‘Communicating and understanding’ learning objectives	Learning objectives addressed communication modalities and situations, and miscommunication
Unit 4 ‘Getting along together at school’ learning objectives	Learning objectives addressed understanding needs versus wants, conflicting needs, offering and accepting help
Unit 5 ‘Is that fair?’ planned objectives	Learning objectives addressed equality and equity-based concepts of fairness; applying fairness concepts to classroom supports, treatment at school
Unit 6 ‘Different ways to make a friendship’ learning objectives	Learning objectives addressed friendship and relationships with classmates
Unit 7 ‘Neurodiversity in our classroom’ learning objectives	Consolidation and reflection unit. Learning objectives involved reviewing past lessons, reflection and planning towards the future

In each case, respondents were asked whether they would *support* the use of the planned programme in primary schools based on these resource components, with three response options: ‘overall yes’, ‘maybe, with changes’ and ‘overall no’. Next, respondents were asked to rate each planned component for how *acceptable* and *useful* they viewed it to be. Acceptability referred to whether the content was respectful and accurate, and usefulness referred to the likelihood of the resource making a positive impact at school. Ratings were collected using a six-point scale with response options ranging from ‘completely unacceptable’ to ‘completely acceptable’, or ‘completely useless’ to ‘very useful’. Finally, in each section, open-ended text boxes were provided as an option for respondents to give detailed feedback.

### Procedure

The survey was approved by the university’s relevant research ethics committee, and consent was obtained from all respondents via an online consent form prior to gaining access to the survey. We circulated the survey on Twitter, Facebook and Reddit and via email. The survey was particularly directed to the neurodivergent community by circulating it via relevant U.K.-based charities, support groups, and social media pages. The survey was also circulated to our professional networks.

### Analysis methods

Respondents were only included in the analyses if they had provided complete ratings for at least one of the key planned resource components included in the survey, in addition to completing the compulsory demographics section. A total of 216 responses were recorded, and 111 were included in the analyses. Out of those who were included, 41% of respondents had completed the full survey. Over 80% of excluded respondents were those who started the survey but did not progress beyond the consent form section.

Quantitative data (support, acceptability and usefulness ratings) were analysed by expressing ratings as a percentage of the total number of responses received for each survey question. Significance testing and statistical comparisons were not conducted as part of this analysis since our goal was to describe community support for the planned content.

Qualitative data (open-ended survey responses) were subject to a simple form of content analysis. Initially, comments were grouped into three categories based on content: positive comments (mention praise or approval), neutral comments (mention general thoughts without making specific suggestions or value judgements) and concerns/suggestions (mention suggestions for improvement or additions, or potential concerns). We focused on the concerns/suggestions for the next part of the analysis. We aimed to find out whether any specific concerns/suggestions were shared across a large proportion of commenters, which might indicate a substantial need for changes to be made to the resource. To do this, we categorised the comments that reported concerns/suggestions for each research component into *topics*, based on whether two or more comments mentioned the same concern/suggestion in relation to a specific resource component. This analysis was completed by the primary author only, and we acknowledge that this may limit the reliability of the results.

### Community involvement

This consultation study was led by a neurodiverse team. As described throughout this article, community involvement was also extensive across other stages of LEANS programme development.

## Results

### Overall support for the planned LEANS resource components

**Figure 1.**
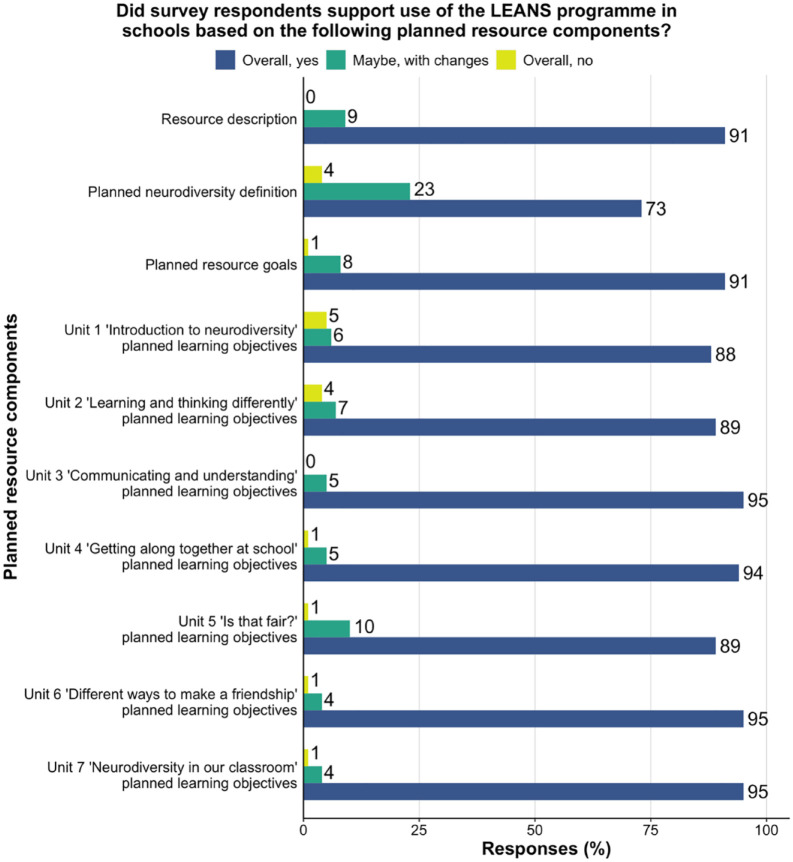
Support for the use of the planned LEANS resource based on key resource components. *Note.* This figure shows the support ratings for the planned LEANS resource components expressed as a percentage of the total number of responses received per component.

Ninety-one per cent of respondents expressed that they would support the use of the LEANS programme based on its description and goals (Figure 1). Four out of the seven educational units (Units 3, 4, 6 and 7) were particularly well received, with 94% or more of respondents expressing support for the learning objectives planned for these units. Compared to this, support was slightly lower for learning objectives in Units 1, 2 and 5 (⩾ 88%). Regarding the definition of neurodiversity shared with respondents, 73% of the sample expressed support and 23% of respondents expressed a desire for changes.

### Views on acceptability and usefulness of the planned LEANS resource components

The ratings for acceptability and usefulness largely aligned with the support ratings. Approximately 90% or more of respondents rated all resource components positively on both acceptability and usefulness. The definition of neurodiversity, and the learning objectives for Units 1, 2 and 5 ranked lowest on both acceptability and usefulness, though even here scores remained high (⩾ 88%). The complete ratings for acceptability and support can be found in Appendix II.

### Open-ended comments on the planned LEANS resource components

For most content shared, a small proportion of respondents left open-ended comments (⩽ 10%). Exceptions were the neurodiversity definition and learning objectives for Unit 1, which received comments from 21% and 14% of respondents, respectively. Comments made across all resource components fell into eight topic categories (Table 3). Most of these were about terminology and ambiguity in explanation of concepts. Although we received multiple comments on a topic, the specific concerns and suggestions were variable, and there was no clear consensus across commenters about desired changes, omissions, or additions. Topics 1 to 5 were raised in relation to multiple resource components and were thus, more generally applicable to the resource. In contrast, topics 6 to 8 were associated with specific resource components, that is the overall resource description, Unit 3 and Unit 6, respectively.

**Table 3.** Concerns and suggestions from open-ended comments about the planned LEANS resource components.

Topics	Comments *n*									
	Resource description	Neurodiversity definition	Resource goals	Unit 1 learning objectives	Unit 2 learning objectives	Unit 3 learning objectives	Unit 4 learning objectives	Unit 5 learning objectives	Unit 6 learning objectives	Unit 7 learning objectives
1. Complicated language or phrasing		8				3		2		
2. Issues with the use of specific terminologies, e.g. ‘neurodivergent’, ‘neurotypical’, ‘label’, ‘behaviour’		6	2	10				2		
3. Ambiguity in the definition of concepts, e.g. neurodiversity, wants and needs, fairness		3	2		2		3			
4. Concerns about othering neurodivergent people				2	2			4		3
5. Suggestion to explicitly refer to disability	2	2								
6. Suggestion to have wider school involvement	2									
7. Suggestion to mention different types of communication						3				
8. Suggestion to consider different social preferences of neurodivergent pupils									3	
**Percentage of total respondents (%)**										
Commented concerns and suggestions	4	21	5	14	2	7	4	10	4	4
Commented positive feedback	0	0	0	8	2	4	1	9	2	1
Commented neutral feedback	1	2	0	8	8	5	5	6	4	1
Did not comment	95	77	95	70	88	84	90	74	90	94

*Note.* This table shows the concerns and suggestions made for the planned LEANS resource components in open-ended comments, and the number of comments mentioning these points for each resource component. It also shows respondents categorised by how they commented on the resource components.

## Discussion

Planned elements of the LEANS programme received a high level of support from respondents to this survey, the majority of whom were neurodivergent. Our small neurodiverse team of researchers and educators successfully produced a resource design that was endorsed by a larger, neurodiverse group of stakeholders, providing validation of our participatory design process. The sample reported high familiarity with the concept of neurodiversity and was largely made up of educators and parents, and was thus well-placed to provide insightful feedback on the resource. This result adds to a growing body of evidence for the utility of participatory design in the development of educational resources (Sjölund et al., 2022).

To date, educational interventions to increase awareness about neurodevelopmental conditions have focused on individual diagnostic groups, perhaps following a tendency for research to be constrained to single diagnoses or occasional comparisons between two diagnoses (Astle et al., 2022; Fletcher-Watson, 2022). However, delivering individual interventions or teacher training separately for each one of multiple forms of neurodivergence has significant resource implications, especially in school systems where teachers already face high levels of workload and time constraints (Kokkinos, 2007). There is also a danger that such approaches may leave out some of the rarer profiles, such as Tourette syndrome, or those which are under-recognised, such as developmental language disorder. Here, we provide evidence for endorsement of an alternative strategy of using the neurodiversity framework to teach about multiple diagnostic categories within a single curriculum, which might be more feasible for implementation in schools.

Following the survey, the LEANS design and planned components were further developed over an eight-month period into a complete version of the resource, used in the LEANS evaluation study (Alcorn et al., 2023). This process involved other forms of consultation, such as feasibility-focused feedback from teachers. The complete resource also took into account open-ended comments from this survey, especially the desirability of precision in language and the need to be fully inclusive. Aside from this, we did not make substantive changes to the programme design following the survey. This is, first, because there were no other consistent, actionable recommendations made by multiple commenters. Second, endorsement of the content presented was so high that any changes in response to a minority would risk the loss of that majority support. Importantly, no comments questioned the underlying ethos or fundamental purpose of the programme.

The small sample size, positive selection bias and homogeneity of the sample are key limitations. The respondents were mostly white, female, and were largely recruited via social media. We did not effectively reach neurodivergent people of colour, and neurodivergent people with disabilities that restrict access to technology – therefore, limiting the generalisability of the survey results. Further along in developing the resource, we did have informal consultations with experts by experience (i.e. people with minority identities) to shape our representation of diversity (gender, ethnicity, disability, culture) in LEANS resource elements, particularly the story characters and illustrations. Overall, views on diversity and representation in the LEANS story content were positive, and minor suggestions were made to improve the representation of specific minority groups.

Furthermore, the majority of neurodivergent respondents included in the survey reported diagnoses of either autism spectrum disorder or Asperger’s syndrome. Other diagnostic groups, and neurotypical people, were under-represented in the sample. Potential reasons for this include the long completion time (est. 15–25 min) and a large amount of text included in the survey, which may have made it less accessible to specific neurodivergent groups, for example, people with ADHD, developmental language disorder, or dyslexia. Time and reading demands may have resulted in the high number of dropouts overall. Alternative methods such as interviews might be needed to accommodate more accessibility needs within the target audience, and future research of this nature should also use advertisement strategies to specifically target marginalised groups.

In conclusion, this study validates the use of a participatory approach to design a primary school teaching programme about neurodiversity. We found that the LEANS programme plan received a high level of support from a neurodiversity-aware, majority-neurodivergent sample. This provided validation to continue with implementing our plans for developing the LEANS programme and provides a preliminary basis for using this approach in the development of future educational and public health resources.

## Supplemental Material

sj-docx-1-aut-10.1177_13623613231211046 – Supplemental material for Short report: Evaluation of wider community support for a neurodiversity teaching programme designed using participatory methods Supplemental material, sj-docx-1-aut-10.1177_13623613231211046 for Short report: Evaluation of wider community support for a neurodiversity teaching programme designed using participatory methods by Reesha Zahir, Alyssa M. Alcorn, Sarah McGeown, Will Mandy, Dinah Aitken, Fergus Murray and Sue Fletcher-Watson in Autism

sj-docx-2-aut-10.1177_13623613231211046 – Supplemental material for Short report: Evaluation of wider community support for a neurodiversity teaching programme designed using participatory methods Supplemental material, sj-docx-2-aut-10.1177_13623613231211046 for Short report: Evaluation of wider community support for a neurodiversity teaching programme designed using participatory methods by Reesha Zahir, Alyssa M. Alcorn, Sarah McGeown, Will Mandy, Dinah Aitken, Fergus Murray and Sue Fletcher-Watson in Autism
