# Supplementary material for: Short report: Evaluation of wider community support for a neurodiversity teaching programme designed using participatory methods
Source: Autism. 2023 Nov 9;28(6):1582–90. doi: 10.1177/13623613231211046 (PMC11134974; doi:10.1177/13623613231211046)
Supplement: sj-docx-1-aut-10.1177_13623613231211046 – Supplemental material for Short report: Evaluation of wider community support for a neurodiversity teaching programme designed using participatory methods [file sj-docx-1-aut-10.1177_13623613231211046.docx]

Appendix I

Complete Community Consultation Survey for the LEANS Educational Resource

LEANS Public Consultation Survey

Start of Block: Welcome

**Welcome to the Learning About Neurodiversity in Schools (LEANS) Project**  
The LEANS project aims to develop a **free**educational resource to educate primary school children about **neurodiversity.**

 When we say **'neurodiversity**' we are talking about the fact that variation between people's brains is a natural part of human life. These variations give rise to differences, that are sometimes associated with diagnoses and labels, like autism, ADHD, dyslexia or dyspraxia.

 By educating children about this, we hope to help create a more empathetic and inclusive classroom environment for all children. 

 In this part of the project, **we are conducting a survey to get the general public's opinion on the LEANS educational materials we have designed.** If you would like to find out more about LEANS and the survey, you may continue to the next page where you will find the participant information sheet giving more details. If you decide to participate in the survey, you will have to complete the consent form on the following page, after which you will be able to access the survey. 

 Going over the **participant information sheet and consent form** will take approximately **5-7 minutes**, and the **survey** itself will take approximately **15-25 minutes** to complete. Your progress is automatically saved, so you will have the option of returning to the survey at a later time (by clicking on the same link you used to get here) if you wish to do so. 


 Please click on the arrow below to continue.

End of Block: Welcome

Start of Block: Participant Information Sheet

**Participant Information Sheet**
 
**Who is this survey for?**
**We are looking for responses from anyone aged 18 or above who is interested in neurodiversity.** While the survey is open to anyone, **we are particularly interested in hearing from people who could be described as neurodivergent** – even if that’s not the word they would use about themselves. Neurodivergent people might have diagnoses like dyslexia, dyspraxia, developmental language disorder, ADHD or autism. They might also self-identify with one of those categories, without having a formal diagnosis.

 **If you are neurotypical**, if you’re not sure if you’re neurodivergent, or if you don’t like the word, please complete the survey anyway – **we want to hear from you too!**


 **What am I being invited to do?**
During this project, our team have been designing a new set of activities and teacher guidance, to help children in mainstream, UK primary schools learn about the concept of neurodiversity. The activities are for a whole class of 8-11 year olds to take part. They have been created by a neurodiverse design team, including researchers and educators.

 We would like to invite members of the public – especially neurodivergent people – to give us their judgement of the **acceptability** and **usefulness** of some elements of the activity pack. We will be asking you about:

- The over-arching goals of the activity pack
- The definitions of neurodiversity we are giving to teachers, and to pupils as part of the materials
- The key points being communicated in the class activities
- The way we are planning to advertise the activity pack
- Any risks to pupils or teachers that we haven’t already identified

We will present you with short written examples, that represent the main ideas and types of content in the classroom activity pack. We will ask you to read through these and rate whether they are acceptable and useful.

 At the end of the survey, you can choose to send us your details, if you also want to take part in a **paid opportunity** to help us examine representation in the materials – such as ethnic, cultural and gender diversity. A small number of people may be contacted later to help us with this.


 **What will happen to my answers?**
 We will gather your ratings and combine them with information from other sources including teachers, and primary school aged children.  We will use the collected feedback to make adjustments to the activity pack.

 This doesn’t mean that your specific comments will necessarily be adopted – we will have to combine information from multiple sources, and not everyone rating the materials will agree. Overall, we are aiming to publish a classroom an activity pack that meets high standards of **acceptability** and **usefulness**, as defined by a neurodiverse community.

 We won’t publish the anonymised responses from the survey in full, but they will be available if someone requests the data for a legitimate reason – for example, if they want to check the accuracy of what we did.  In this case, we would remove any open-ended responses (because they can inadvertently reveal details about the people responding) and we would also remove some personal details like age and gender. The rest of the data would then be shared as a spreadsheet with the person who made the request.


 **How long will the survey take? Can I come back later?**
 The whole survey should take **approximately 15-25 minutes** to complete. For people who may require more reading time, we estimate it might take approximately 40-50 minutes.

 If you are not able to complete the survey in one go you can **return to it later** by clicking on the link you used to get here, or using the following link [<https://edinburgh.eu.qualtrics.com/jfe/form/SV_en9JghX0BKf7zdb>]. Please note you will need to make sure you are using the same computer/device and the same browser to access the survey if you come back to it later.


 **Who is conducting the research?**
 This survey is being carried out by researchers at the University of Edinburgh and has received ethical approval from the Moray House School of Education and Sport Ethics Committee.

 If you would like further information or have any questions about this survey, please contact [Author] by email.


 **Do I have to take part?**
 No, you do not have to take part in this survey, but starting this survey indicates a willingness for your data to be used by the research team.

 If you want to withdraw your data there are two ways to do that. First, at the end of the survey there will be a question which gives you the opportunity to withdraw your answers if you change your mind about taking part. Alternatively, if you want to quit and withdraw halfway through you can contact the research team to request that your data are removed. We will ask you to provide a personal "keyword", when you start the survey, to help us identify your data and remove it from our files. You do not have to provide a reason for withdrawing. 

 The option to withdraw your data will remain open until everyone's survey responses have been collected. After this time the data from every participant will be entered into an analysis, and it will no longer be possible to remove individual responses. 


 **What should I do if I want to make a complaint?**
 Any concerns should be directed in the first instance to [Principal Investigator] by email or by phone/text.

 If you have concerns which you believe have not been addressed then complaints can be made to Head of the Division of Psychiatry at the University of Edinburgh, [Name of Head of Division]. A complaint form can be found here [URL].


 **How do I participate?**
 If you are interested in participating in this survey, please click the arrow below to continue to the next page and complete the consent form. You will then be able to access the survey.

End of Block: Participant Information Sheet

Start of Block: Consent form

**Consent Form**
 
Please answer the following in order to participate in the survey:

|  | Yes | No |
| --- | --- | --- |
| I understand that I can contact [Author] by email if I have any questions about this survey or if I wish to withdraw my data |  |  |
| I confirm that I am aged 18 or older |  |  |
| I understand that I can skip individual questions or quit the survey at any point without giving a reason |  |  |
| I understand that anonymised data will be combined with data from other participants, for analysis and publication |  |  |
| I consent to take part in this survey |  |  |

**Key Word**

 Before you begin the survey, please create a keyword that is unique to you. This keyword will only be used for the purpose of identifying your data if you decide that you want to withdraw from the survey. It will not be used for any other purpose.

 Your keyword should be: your favourite food, followed by your favourite place, followed by your favourite colour. 

 For example: My favourite food is chocolate, my favourite place is the garden and my favourite colour is orange. My keyword would be: chocolategardenorange.
 
Please enter your keyword here:

________________________________________________________________

End of Block: Consent form

Start of Block: Survey Welcome

**Welcome to the LEANS Public Consultation Survey**
 
Thank you for choosing to take part in this survey to help us improve the LEANS educational materials, we really appreciate your contribution to our project.

 We estimate that this survey might take approximately **15-25 minutes** to complete. For people who may require more reading time, we estimate it might take approximately 40-50 minutes. If you are not able to complete the survey in one go, **your progress is automatically saved, and you can return to the survey at a later time by clicking the link you used to get here.** Please note that you will need to use the same computer/device and the same browser to access the survey at a later time. 

 Please also be assured that your answers will be recorded anonymously and kept with strict confidentiality.  
 
Please click the right arrow below to continue.

End of Block: Survey Welcome

Start of Block: LEANS Information

Before you begin...

We would like to summarise some key information about the LEANS project to help you navigate through the survey*.* Our project, **Learning about Neurodiversity at School (LEANS),** aims to to develop a **freely available** resource to teach primary school children about **neurodiversity**. When we say **'neurodiversity**', we are talking about the fact that variation between people’s brains is a natural part of human life. These variations give rise to differences, that are sometimes associated with diagnoses and labels, like autism, ADHD, dyslexia or dyspraxia. In teaching about neurodiversity, we are focusing specifically on **similarities and differences in how we all learn and enjoy friendships**, and the **challenges we can encounter with these aspects in school life**. Overall, we hope that this resource could potentially help create a more empathetic and inclusive school environment for all children. The LEANS educational materials will include teacher guidance and classroom activities. We will present you with short written examples that represent the main ideas and types of content in these materials, and ask your opinion about the **acceptability** and **usefulness** of the content. 
   At the end of the survey, you will also have the option to send us your details if you would like to take part in a **paid opportunity**to help us examine representation in the materials - such as ethnic, cultural and gender diversity. You may click the right arrow below when you are ready to begin the survey.

End of Block: LEANS Information

Start of Block: Part 1: Demographics

Part 1: **Demographic Information**

 In this section, we will ask you a few questions about yourself in order to help us better understand what people from different backgrounds may think about the LEANS activity pack. **Please be assured that your responses will be recorded anonymously and kept with strict confidentiality.**

1. Where are you currently based?

- England
- Northern Ireland
- Scotland
- Wales
- Ireland
- Other (please specify) ________________________________________________

2. What is your age in years?

________________________________________________________________

3. Do you have any of the following diagnoses, or do you otherwise identify as neurodivergent?

|  | Yes, diagnosed | Yes, self-identified | Suspected, but not sure | No |
| --- | --- | --- | --- | --- |
| Autism / Autism Spectrum Disorder (ASD) / Autism Spectrum Condition (ASC) |  |  |  |  |
| Attention Deficit Disorder (ADD)/ Attention Deficit Hyperactivity Disorder (ADHD) |  |  |  |  |
| Asperger's Syndrome |  |  |  |  |
| Pervasive Developmental Disorder – Not Otherwise Specified (PDD-NOS) |  |  |  |  |
| Dyslexia |  |  |  |  |
| Dyspraxia |  |  |  |  |
| Developmental Language Disorder |  |  |  |  |
| Other (please specify) |  |  |  |  |

4. Do you have any immediate family members (spouse, sibling, co-parent, children, parents) who are neurodivergent? *Please simply select all the diagnostic categories that apply to one or more people in your family – we don’t need to know who, or how many of each.*

- Autism / Autism Spectrum Disorder (ASD) / Autism Spectrum Condition (ASC)
- Attention Deficit Disorder (ADD) / Attention Deficit Hyperactivity Disorder (ADHD)
- Asperger's Syndrome
- Pervasive Developmental Disorder-Not Otherwise Specifies (PDD-NOS)
- Dyslexia
- Dyspraxia
- Developmental Language Disorder
- Other (please specify) ________________________________________________
- I do not have immediate family members who are neurodivergent

5. Are you a parent or carer of a child aged under 18?

- Yes
- No
- Other (please specify) ________________________________________________

6. Do you have more than 2 years of experience in any of the following professions?

- Teacher
- Learning Support/ Classroom Assistant
- Educational Psychologist
- Specialist Teacher
- Other educational role (please specify) ________________________________________________
- I do not have experience in the professions listed

7. What is your gender?

- Female
- Male
- Non-binary
- Prefer not to say
- Other (please specify) ________________________________________________

8. What is your ethnicity?
*Please note that the categories listed were taken from the standard UK consensus.*

- Asian - Bangladeshi
- Asian - Chinese
- Asian - Indian
- Asian - Pakistani
- Any other Asian background (please specify) ________________________________________________
- Arab
- Black - African
- Black - Caribbean
- Any other Black background (please specify) ________________________________________________
- White - English / Welsh / Scottish / Northern Irish / British
- White - Irish
- White - Gypsy or Irish Traveller
- Any other White background (please specify) ________________________________________________
- Mixed - White and Asian
- Mixed - White and Black African
- Mixed - White and Black Caribbean
- Any other Mixed background (please specify) ________________________________________________
- Any other ethnic group (please specify) ________________________________________________
- Prefer not to say

End of Block: Part 1: Demographics

Start of Block: Part 2: Website Information

Part 2:**Web Information about the LEANS Resource Pack**

 In this section, we will ask you what you think of the online information about the LEANS resource pack. 

 Below is a draft of the opening paragraphs from the website where we plan to provide information about the classroom activity pack. The website will be the main place that teachers can come to download the materials, and we will also have information for pupils and their parents. There will be further information on the site, but the text below will be the first thing that people will see on the website: 
 
**What is the LEANS Resource Pack about?**
The Learning About Neurodiversity at Schools (LEANS) resource pack is a free educational resource about *neurodiversity*- the variation between people's brains which make us think and process information in different ways. These differences are sometimes associated with diagnoses such as Autism, ADHD, Dyslexia or Dyspraxia. This resource is designed for delivery in mainstream primary schools, for children aged 8-11 years. 

 With the LEANS resource pack, we want to help all children understand that neurodiversity is a natural part of human life, and that it is okay for people to have different experiences and needs when it comes to learning, playing and socialising at school. By teaching kids to embrace these differences, we hope to create a more empathetic and inclusive classroom environment, aiming to provide the best conditions for all children to thrive and succeed in school. The materials in the LEANS resource pack are designed to convey key concepts about neurodiversity to the whole classroom, including neurotypical and neurodivergent students. Please note that this is purely an educational resource, not intended to be substituted for neurodevelopmental specialist advice or statutory guidance.

9. Based on the above information, does the LEANS resource pack seem **acceptable**and **useful**enough that you would want to find out more about the contents and materials?

A resource that is **acceptable**should use respectful language and methods to convey its message, and contain accurate information - even though it may be simplified for a young audience. 
 A resource that is **useful**to teachers and pupils should contain relevant content that stands a good chance of having a positive impact in a school context.

| ***Acceptability*** | ***Usefulness*** |
| --- | --- |
| - Completely unacceptable | - Completely useless |
| - Unacceptable | - Useless |
| - Needs improvement | - Needs improvement |
| - Okay | - Okay |
| - Acceptable | - Useful |
| - Completely acceptable | - Very useful |

10. Assuming all other aspects were okay, would you feel supportive of using the LEANS resource pack to teach about neurodiversity in mainstream primary schools, based on this web description?

- On the whole, yes
- On the whole, no
- Maybe, with changes

10A. If you answered 'maybe, with changes', please give details here

________________________________________________________________

________________________________________________________________

________________________________________________________________

________________________________________________________________

________________________________________________________________

End of Block: Part 2: Website Information

Start of Block: Part 3: LEANS Resource Pack Format

Part 3: **LEANS Resource Pack Format**
  
In this section, we will ask you what you think about the planned **format** of the LEANS resource pack, and our teaching strategy. First, we ask you to please read this short information about the resource pack.

The LEANS resource pack will be available for free online. It will be made up of a **teacher handbook**plus **downloadable materials.** The **handbook**is written for mainstream primary school teachers at all levels of experience with neurodiversity, including those encountering topic for the first time. It includes:

- An introduction to the goals and the purpose of the resource pack
- Information about the concept of neurodiversity, and related vocabulary like neurodivergence (see the definition we are using in survey section 4)
- Guidance for safely and ethically teaching this topic
- How-to-instructions for delivering the LEANS resources in class
- Guidance to support conversations with parents and students about neurodiversity

Within the teacher handbook, LEANS resources are divided into **seven units**, each focusing on a single topic and structured around **Key Points.**These points state the core concepts in each unit that students should understand upon its completion. Section 6 of this survey present the Key Points and ask for your feedback. 
   In each unit, **hands-on activities**are integrated with **storytelling**content about a fictional, neurodiverse classroom of students who are also learning about ideas in the LEANS resource pack.
   Stories may introduce new ideas that are then explored in a hands-on activity, or may follow an activity to help "make sense" of those direct experiences. Through storytelling and fictional characters, we can illustrate neurodiversity and neurodivergence in a concrete and specific way, but while reducing risks of pupils feeling singled out or judged where LEANS materials touch on real issues in their class. Our characters will represent a range of school experiences, strengths and challenges, and will have different diagnostic statuses (diagnosed neurodivergent, differences but no diagnosis, and neurotypical). 
   The LEANS **downloadable materials**include instructions, stories and illustrations and other items needed to deliver the stories and activities in the classroom. Again, the handbook and all the materials will be free.

11. Does structuring the LEANS resources as a teacher handbook and downloadable materials seem acceptable and useful?

An **acceptable**format for the resource should convey information accurately using respectful methods and language. 
 A **useful** format of the resource for teachers and pupils should include relevant content and have a high chance of making a positive impact in a school context.

| ***Acceptability*** | ***Usefulness*** |
| --- | --- |
| - Completely unacceptable | - Completely useless |
| - Unacceptable | - Useless |
| - Needs improvement | - Needs improvement |
| - Okay | - Okay |
| - Acceptable | - Useful |
| - Completely acceptable | - Very useful |

12. Does teaching neurodiversity concepts through hands-on activities plus storytelling seem acceptable and useful?

An **acceptable**format for the resource should convey information accurately using respectful methods and language. 
 A **useful** format of the resource for teachers and pupils should include relevant content and have a high chance of making a positive impact in a school context.

| ***Acceptability*** | ***Usefulness*** |
| --- | --- |
| - Completely unacceptable | - Completely useless |
| - Unacceptable | - Useless |
| - Needs improvement | - Needs improvement |
| - Okay | - Okay |
| - Acceptable | - Useful |
| - Completely acceptable | - Very useful |

End of Block: Part 3: LEANS Resource Pack Format

Start of Block: Part 4: Neurodiversity Definitions

Part 4: **Definition of Neurodiversity used in the LEANS Resource Pack**

 In this section, we will ask you what you think about the **definition**of neurodiversity we have used in the LEANS resource pack.

13. How would you rate your familiarity with the concept of neurodiversity?

- Very familiar
- Somewhat familiar
- Mostly unfamiliar
- Completely unfamiliar

14. The following is the definition of neurodiversity **for teachers** included in the 'About Neurodiversity' section of the LEANS teacher handbook resource. We aim for this definition to be accessible to teachers with who are completely new to the topic, as well as those who are already familiar with it. Is this definition (below) **acceptable** and **useful?** 
    *"Neurodiversity is the fact that all human beings vary in the way our brains work. We take in information in different ways, we process it in different ways, and thus we behave in different ways. Neurodiversity is a property of the entire human race - each individual person is different from the next. Neurodiversity also gives rise to categorical differences between people. These categorical differences in brain processes, and therefore in experiences and behaviour, underpin diagnostic labels such as autism or dyspraxia"*

A definition that is **acceptable** should use respectful language and contain accurate information - even though it may have been simplified for a young audience.

A definition that is **useful**to teachers and pupils should contain relevant content that stands a good chance of having a positive impact in a school context.

| ***Acceptability*** | ***Usefulness*** |
| --- | --- |
| - Completely unacceptable | - Completely useless |
| - Unacceptable | - Useless |
| - Needs improvement | - Needs improvement |
| - Okay | - Okay |
| - Acceptable | - Useful |
| - Completely acceptable | - Very useful |

15. Assuming all other aspects were okay, would you feel supportive of primary school resources which used this definition of neurodiversity?

- On the whole, yes
- On the whole, no
- Maybe, with changes

15A. If you answered 'maybe, with changes', please give details here

________________________________________________________________

________________________________________________________________

________________________________________________________________

________________________________________________________________

________________________________________________________________

End of Block: Part 4: Neurodiversity Definitions

Start of Block: Part 5: Goals

Part 5:**Goals of using the LEANS Resource Pack in mainstream primary schools**

 In this section, we will ask you to think about the **goals** of using the LEANS resource pack to teach about neurodiversity in mainstream primary schools. All of these states goals apply both to pupils, and to school staff members involved in delivering the resource pack. 
 
Please score each of the following project goals (listed in questions 16-18) for how acceptable and useful they are.

Goals that are **acceptable** should be meaningful and use respectful language.

Goals that are **useful** to teachers and pupils should be relevant and have a high chance of making a positive impact in a school context.

16. To increase knowledge of neurodiversity terms and concepts

| ***Acceptability*** | ***Usefulness*** |
| --- | --- |
| - Completely unacceptable | - Completely useless |
| - Unacceptable | - Useless |
| - Needs improvement | - Needs improvement |
| - Okay | - Okay |
| - Acceptable | - Useful |
| - Completely acceptable | - Very useful |

17. To increase individuals' positive and inclusive **actions** within the school community

| ***Acceptability*** | ***Usefulness*** |
| --- | --- |
| - Completely unacceptable | - Completely useless |
| - Unacceptable | - Useless |
| - Needs improvement | - Needs improvement |
| - Okay | - Okay |
| - Acceptable | - Useful |
| - Completely acceptable | - Very useful |

18. To create more positive **attitudes**towards neurodiversity and neurodivergent people, following participation in the LEANS curriculum

| ***Acceptability*** | ***Usefulness*** |
| --- | --- |
| - Completely unacceptable | - Completely useless |
| - Unacceptable | - Useless |
| - Needs improvement | - Needs improvement |
| - Okay | - Okay |
| - Acceptable | - Useful |
| - Completely acceptable | - Very useful |

19. Assuming all other aspects were okay, would you feel supportive of primary school resources with these goals?

- On the whole, yes
- On the whole, no
- Maybe, with changes

19A. If you answered 'maybe, with changes', please give details here

________________________________________________________________

________________________________________________________________

________________________________________________________________

________________________________________________________________

________________________________________________________________

End of Block: Part 5: Goals

Start of Block: Part 6: Key Points (Units 1-2)

Part 6: **Key content points used to structure the LEANS content**

 The resource pack is structured into seven units, with the central ideas of each unit summarised by “key points”. Pupils and teachers then explore the unit's key points through one or more interactive activities. In this section, we will ask you what you think about the **acceptability** and **usefulness** of these **key points.**

Key points that are **acceptable**should use respectful language and contain accurate information - even though it may have been simplified for a young audience.

Key points that are **useful**to teachers and pupils should contain relevant content that stands a good chance of having a positive impact in a school context.

**LEANS Resource Pack Unit 1 Key Points**

 Resource content for LEANS **Unit 1, 'Introduction to Neurodiversity'** is structured around the following key points:   
  
**1.1 Neurodiversity means that we are all different in how we think, feel and learn, because our brains are different.** Neurodiversity includes everyone, because everyone has a brain!
  
1.2 Differences between people's brains can be little differences, or big differences. Most people have brains that are only a little different from one another, and they are called neurotypical. However, some people's brains have big differences compared to neurotypical brains. People with "more different" brains like these are called neurodivergent. 

 **1.3 Neurodivergent people are also different from each other.**Sometimes, we use labels to describe patterns of differences, such as Dyslaxia, Dyspraxia, ADHD or Autism. Neurodivergent people might have one of these labels, or several, or none at all.

 **1.4 How I choose to treat other people in class, like when we're working in a group, can make a big difference to how they feel and how their school day goes.**

  20. Please rate these key points for how **acceptable** and **useful** they are as part of a unit that introduces neurodiversity.

| ***Acceptability*** | ***Usefulness*** |
| --- | --- |
| - Completely unacceptable | - Completely useless |
| - Unacceptable | - Useless |
| - Needs improvement | - Needs improvement |
| - Okay | - Okay |
| - Acceptable | - Useful |
| - Completely acceptable | - Very useful |

21. If you have comments about the acceptability or usefulness of specific Unit 1 key points, please give details here.

________________________________________________________________

________________________________________________________________

________________________________________________________________

________________________________________________________________

________________________________________________________________

22. Assuming all other aspects were okay, would you feel supportive of primary school resources that used these key points to introduce neurodiversity?

- On the whole, yes
- On the whole, no
- Maybe, with changes

22A. If you answered 'maybe, with changes', please give details here

________________________________________________________________

________________________________________________________________

________________________________________________________________

________________________________________________________________

________________________________________________________________

**LEANS Resource Pack Unit 2 Key Points**
 Resource content for LEANS **Unit 2, 'Learning and Thinking Differently'** is structured around the following key points: 
  
**2.1 We all learn and think differently.** "Learning and thinking" including things like remembering, paying attention, writing and coming up with ideas. 

 **2.2 Just like neurodivergent people have "more different" brains, so the differences in how they think and learn are bigger too.**They may find some things very easy, very hard, or just *different*compared to neurotypical people.

 **2.3 Even when my class all does the same activity, other people are not always having the same experience that I am.**

 **2.4 What I need in order to focus and learn at school may be different to what other people need.**Having different learning needs is okay. 

 23. Please rate these key points for how **acceptable** and **useful**they are as part of a unit about differences in learning and thinking.

| ***Acceptability*** | ***Usefulness*** |
| --- | --- |
| - Completely unacceptable | - Completely useless |
| - Unacceptable | - Useless |
| - Needs improvement | - Needs improvement |
| - Okay | - Okay |
| - Acceptable | - Useful |
| - Completely acceptable | - Very useful |

24. If you have comments about the acceptability or usefulness of specific Unit 2 key points, please give details here.

________________________________________________________________

________________________________________________________________

________________________________________________________________

________________________________________________________________

________________________________________________________________

25. Assuming all other aspects were okay, would you feel supportive of primary school resources that used these key points to teach pupils about differences in learning and thinking?

- On the whole, yes
- On the whole, no
- Maybe, with changes

25A. If you answered 'maybe, with changes', please give details here

________________________________________________________________

________________________________________________________________

________________________________________________________________

________________________________________________________________

________________________________________________________________

End of Block: Part 6: Key Points (Units 1-2)

Start of Block: Part 6: Key Points (Units 3-4)

**LEANS Resource Pack Unit 3 Key Points**
   Resource content for LEANS **Unit 3, 'Communication and Understanding'**is structured around the following key points: 
  
**3.1 We can use different ways to share information, thoughts or feelings.** All ways of communicating are useful and important, even if those ways might not be the same ones the people around me are using.

 **3.2 Different people may find it easier or harder to communicate in certain ways.**

 **3.3 We may not be able to automatically tell what other people are thinking or trying to communicate, if they communicate in a different way than we do.**If we make assumptions, we might get it wrong. It can be okay to ask people for more information.

26. Please rate these key points for how **acceptable**and **useful**they are as part of a unit about communication and neurodiversity.

| ***Acceptability*** | ***Usefulness*** |
| --- | --- |
| - Completely unacceptable | - Completely useless |
| - Unacceptable | - Useless |
| - Needs improvement | - Needs improvement |
| - Okay | - Okay |
| - Acceptable | - Useful |
| - Completely acceptable | - Very useful |

27. If you have comments about the acceptability or usefulness of specific Unit 3 key points, please give details here.

________________________________________________________________

________________________________________________________________

________________________________________________________________

________________________________________________________________

________________________________________________________________

28. Assuming all other aspects were okay, would you feel supportive of primary school resources that used these key points to teach pupils about communication and neurodiversity?

- On the whole, yes
- On the whole, no
- Maybe, with changes

28A. If you answered 'maybe, with changes', please give details here

________________________________________________________________

________________________________________________________________

________________________________________________________________

________________________________________________________________

________________________________________________________________

**LEANS Resource Pack Unit 4 Key Points**
 Resource content for LEANS **Unit 4, 'Getting Along at School'**is structured around the following key points: 
  
4.1 *Needs* and *wants* are not the same. Just because I really want something doesn't make it a need.
  
**4.2 What I need may be different than what other people need, and that is okay.**Everyone has things they need to thrive at school and in their life. 
  
**4.3 Sometimes, people may need opposite things (conflicting needs). That doesn't mean that either person is wrong.**We may need to compromise about what happens in those situations to try to help everyone the best we can. 

 29. Please rate these key points for how **acceptable** and **useful** they are as part of a unit that focuses on navigating different needs and wants at school.

| ***Acceptability*** | ***Usefulness*** |
| --- | --- |
| - Completely unacceptable | - Completely useless |
| - Unacceptable | - Useless |
| - Needs improvement | - Needs improvement |
| - Okay | - Okay |
| - Acceptable | - Useful |
| - Completely acceptable | - Very useful |

30. If you have comments about the acceptability or usefulness of specific Unit 4 key points, please give details here.

________________________________________________________________

________________________________________________________________

________________________________________________________________

________________________________________________________________

________________________________________________________________

31. Assuming all other aspects were okay, would you feel supportive of primary school resources that used these key points to teach pupils about navigating different needs and wants at school?

- On the whole, yes
- On the whole, no
- Maybe, with changes

31A. If you answered 'maybe, with changes', please give details here

________________________________________________________________

________________________________________________________________

________________________________________________________________

________________________________________________________________

________________________________________________________________

End of Block: Part 6: Key Points (Units 3-4)

Start of Block: Part 6: Key Points (Units 5-6)

**LEANS Resource Pack Unit 5 Key Points**
 Resource content for LEANS **Unit 5, 'Is that fair?'**is structured around the following key points: 

 **5.1 Fairness in school isn't always about being treated the same or getting the same things.**
  
**5.2 Sometimes, it can be fair for people to *get*or *do*different things than their classmates, because they have different needs.**Being treated fairly helps all of us to do our best at school. 
  
**5.3 Due to their learning and thinking, some neurodivergent students may do things differently in the classroom.**What helps one person may not help another - neurodivergent people can be very different too.
 32. Please rate these key points for how **acceptable** and **useful** they are as part of a unit about fairness in the classroom.

| ***Acceptability*** | ***Usefulness*** |
| --- | --- |
| - Completely unacceptable | - Completely useless |
| - Unacceptable | - Useless |
| - Needs improvement | - Needs improvement |
| - Okay | - Okay |
| - Acceptable | - Useful |
| - Completely acceptable | - Very useful |

33. If you have comments about the acceptability or usefulness of specific Unit 5 key points, please give details here.

________________________________________________________________

________________________________________________________________

________________________________________________________________

________________________________________________________________

________________________________________________________________

34. Assuming all other aspects were okay, would you feel supportive of primary school resources that used these key points to teach pupils about fairness in the classroom?

- On the whole, yes
- On the whole, no
- Maybe, with changes

34A. If you answered 'maybe, with changes', please give details here

________________________________________________________________

________________________________________________________________

________________________________________________________________

________________________________________________________________

________________________________________________________________

**LEANS Resource Pack Unit 6 Key Points**
 Resource content for LEANS **Unit 6, 'Different Ways to Make a Friendship?'**is structured around the following key points: 
  
**6.1 There are many ways to be a good friend or classmate.** We can also be different in the types of friendships we want to have. The same things may not be equally important to every person. 
  
**6.2 No friendship is perfect all the time.**You can still be friends with someone even if you have disagreements, don't always want to do the same things, spend time apart, or are different in other ways. 
  
**6.3 Trying to think about what another person needs or wants, not only what I want, is an important part of being a friend.**I need to remember that other people may need or want different things than I do.

35. Please rate these key points for how **acceptable** and **useful** they are as part of a unit about neurodiversity and friendship.

| ***Acceptability*** | ***Usefulness*** |
| --- | --- |
| - Completely unacceptable | - Completely useless |
| - Unacceptable | - Useless |
| - Needs improvement | - Needs improvement |
| - Okay | - Okay |
| - Acceptable | - Useful |
| - Completely acceptable | - Very useful |

36. If you have comments about the acceptability or usefulness of specific Unit 6 key points, please give details here.

________________________________________________________________

________________________________________________________________

________________________________________________________________

________________________________________________________________

________________________________________________________________

37. Assuming all other aspects were okay, would you feel supportive of primary school resources that use these key points to teach pupils about neurodiversity and friendship.

- On the whole, yes
- On the whole, no
- Maybe, with changes

37A. If you answered 'maybe, with changes', please give details here

________________________________________________________________

________________________________________________________________

________________________________________________________________

________________________________________________________________

________________________________________________________________

End of Block: Part 6: Key Points (Units 5-6)

Start of Block: Part 6: Key Points (Unit 7)

**LEANS Resource Pack Unit 7 Key Points**
 Resource content for LEANS **Unit 7, 'Neurodiversity in Our Classroom'**is structured around the following key points: 
  
**7.1 Learning about differences between types of brains helps us understand how these differences affect people's lives, and that those differences are okay.**
  
**7.2 As individuals and as a class, we choose how we treat each other and how we act toward people who seem different.**Those actions really matter to how people feel and learn at school. We can choose to be kind, respectful, and to listen to others. 
  
**7.3 The way our actions affect other people and ourselves can be complex.**There won't always be a "right answer" that will give *everyone*what they need or want, but by working together we can make things better.

38. Please rate these key points for how **acceptable** and **useful** they are as part of a unit promoting inclusive actions and attitudes about neurodiversity.

| ***Acceptability*** | ***Usefulness*** |
| --- | --- |
| - Completely unacceptable | - Completely useless |
| - Unacceptable | - Useless |
| - Needs improvement | - Needs improvement |
| - Okay | - Okay |
| - Acceptable | - Useful |
| - Completely acceptable | - Very useful |

39. If you have comments about the acceptability or usefulness of specific Unit 7 key points, please give details here.

________________________________________________________________

________________________________________________________________

________________________________________________________________

________________________________________________________________

________________________________________________________________

40. Assuming all other aspects were okay, would you feel supportive of primary school resources that used these key points to promote inclusive actions and attitudes about neurodiversity.

- On the whole, yes
- On the whole, no
- Maybe, with changes

40A. If you answered 'maybe, with changes', please give details here

________________________________________________________________

________________________________________________________________

________________________________________________________________

________________________________________________________________

________________________________________________________________

End of Block: Part 6: Key Points (Unit 7)

Start of Block: Part 7: Risks

Part 7: **Risks**

In this section we will ask you what you think about **potential risks** which may come from using the LEANS activity pack. 
 We acknowledge that neurodiversity is a sensitive topic to explore in classrooms with young students, some of whom could have neurodivergent diagnoses, or be neurodivergent and undiagnosed. Therefore, we are taking every step to identify and protect against potential negative impacts which may arise. Potential risks we have identified include:

- Children feeling singled out or embarrassed by the activities
- Children experiencing victimisation as a result of taking part in the activities
- Children experiencing realisations about themselves that they are not equipped to deal with
- Parents feelings that the topics being discussed in class are inappropriate for this age-group
- Teachers or children unintentionally reinforcing negative views of difference, including stigma or patronising attitudes

41. Are there are other likely risks to pupils that you think we need to consider? Please include details if, for example, you think these risks are relevant to specific sub-groups of pupils.

________________________________________________________________

________________________________________________________________

________________________________________________________________

________________________________________________________________

________________________________________________________________

42. Are there risks to anyone else involved – e.g. parents, siblings, school staff members– that you think we need to consider?

________________________________________________________________

________________________________________________________________

________________________________________________________________

________________________________________________________________

________________________________________________________________

End of Block: Part 7: Risks

Start of Block: Survey end

**Thank you so much!**

 Thank you for taking the time to help us create the best possible resource to educate children about neurodiversity. If you have any other comments to share please do so here:

________________________________________________________________

________________________________________________________________

________________________________________________________________

________________________________________________________________

________________________________________________________________

In addition, we would love to hear from you if you are interested in a **paid opportunity** to help us check representation in the final resource. We want to make sure that the materials we produce have appropriate representation of aspects such as cultural, ethnic, gender and disability diversity. If you have relevant lived experience and want to contribute to this please **open the link below in a new window** and add your details in the new form: 

 Representation Consultation Form 

Finally, a reminder that if you would like further information or have any questions about this survey, please contact [Author] by email.

End of Block: Survey end
