# Supplementary material for: Short report: Evaluation of wider community support for a neurodiversity teaching programme designed using participatory methods
Source: Autism. 2023 Nov 9;28(6):1582–90. doi: 10.1177/13623613231211046 (PMC11134974; doi:10.1177/13623613231211046)
Supplement: sj-docx-2-aut-10.1177_13623613231211046 – Supplemental material for Short report: Evaluation of wider community support for a neurodiversity teaching programme designed using participatory methods [file sj-docx-2-aut-10.1177_13623613231211046.docx]

**Appendix III**

*Acceptability and Usefulness Ratings for the planned LEANS Resource Components*

| **Acceptability Ratings** | **Respondents *n*** | | | | | | | | | | | |
| --- | --- | --- | --- | --- | --- | --- | --- | --- | --- | --- | --- | --- |
|  | Resource description | Planned ND definition | Planned Goal 1 | Planned Goal 2 | Planned Goal 3 | Unit 1 learning objectives | Unit 2 learning objectives | Unit 3 learning objectives | Unit 4 learning objectives | Unit 5 learning objectives | Unit 6 learning objectives | Unit 7 learning objectives |
| Completely unacceptable | 1 | 2 | 0 | 1 | 0 | 2 | 1 | 1 | 1 | 1 | 1 | 0 |
| Unacceptable | 0 | 2 | 0 | 1 | 1 | 3 | 2 | 0 | 1 | 0 | 0 | 1 |
| Needs improvement | 1 | 17 | 2 | 3 | 1 | 5 | 2 | 3 | 2 | 6 | 0 | 2 |
| Okay | 6 | 18 | 3 | 2 | 6 | 3 | 6 | 5 | 7 | 5 | 3 | 3 |
| Acceptable | 37 | 34 | 19 | 23 | 21 | 30 | 26 | 18 | 33 | 23 | 25 | 24 |
| Completely acceptable | 59 | 36 | 73 | 65 | 68 | 41 | 47 | 57 | 41 | 48 | 53 | 49 |
| Total respondents | 104 | 99 | 97 | 95 | 97 | 84 | 84 | 84 | 85 | 83 | 82 | 79 |
| **Usefulness Ratings** |  |  |  |  |  |  |  |  |  |  |  |  |
| Completely useless | 0 | 1 | 0 | 0 | 0 | 3 | 1 | 1 | 1 | 1 | 1 | 0 |
| Useless | 0 | 0 | 0 | 1 | 1 | 1 | 3 | 0 | 1 | 0 | 0 | 1 |
| Needs improvement | 5 | 14 | 2 | 5 | 2 | 5 | 2 | 1 | 3 | 4 | 0 | 1 |
| Okay | 8 | 16 | 8 | 5 | 7 | 4 | 4 | 6 | 8 | 8 | 5 | 3 |
| Useful | 36 | 34 | 19 | 19 | 18 | 13 | 29 | 20 | 31 | 17 | 26 | 24 |
| Completely useful | 57 | 33 | 64 | 64 | 68 | 42 | 45 | 57 | 41 | 52 | 50 | 50 |
| Total respondents | 106 | 98 | 93 | 94 | 96 | 85 | 84 | 85 | 85 | 82 | 82 | 79 |

ND: Neurodiversity.
